# Integrating nitric oxide into salicylic acid and jasmonic acid/ ethylene plant defense pathways

**DOI:** 10.3389/fpls.2013.00215

**Published:** 2013-06-27

**Authors:** Luis A. J. Mur, Elena Prats, Sandra Pierre, Michael A. Hall, Kim H. Hebelstrup

**Affiliations:** ^1^Molecular Plant Pathology Group, Institute of Environmental and Rural Science, Aberystwyth UniversityAberystwyth, UK; ^2^Institute for Sustainable Agriculture, Spanish National Research CouncilCórdoba, Spain; ^3^Section of Crop Genetics and Biotechnology, Department of Molecular Biology and Genetics Aarhus UniversitySlagelse, Denmark

**Keywords:** nitric oxide, salicylic acid, jasmonic acid, ethylenes, pathogens, resistance mechanisms, signaling pathways

## Abstract

Plant defense against pests and pathogens is known to be conferred by either salicylic acid (SA) or jasmonic acid (JA)/ethylene (ET) pathways, depending on infection or herbivore-grazing strategy. It is well attested that SA and JA/ET pathways are mutually antagonistic allowing defense responses to be tailored to particular biotic stresses. Nitric oxide (NO) has emerged as a major signal influencing resistance mediated by both signaling pathways but no attempt has been made to integrate NO into established SA/JA/ET interactions. NO has been shown to act as an inducer or suppressor of signaling along each pathway. NO will initiate SA biosynthesis and nitrosylate key cysteines on TGA-class transcription factors to aid in the initiation of SA-dependent gene expression. Against this, *S*-nitrosylation of NONEXPRESSOR OF PATHOGENESIS-RELATED PROTEINS1 (NPR1) will promote the NPR1 oligomerization within the cytoplasm to reduce TGA activation. In JA biosynthesis, NO will initiate the expression of JA biosynthetic enzymes, presumably to over-come any antagonistic effects of SA on JA-mediated transcription. NO will also initiate the expression of ET biosynthetic genes but a suppressive role is also observed in the *S*-nitrosylation and inhibition of *S*-adenosylmethionine transferases which provides methyl groups for ET production. Based on these data a model for NO action is proposed but we have also highlighted the need to understand when and how inductive and suppressive steps are used.

## INTRODUCTION

**FIGURE 1:**
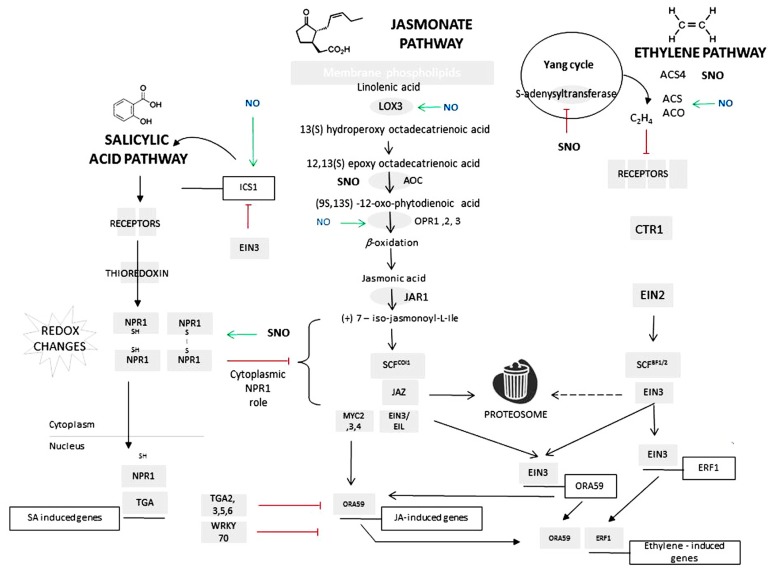
**The impact of nitric oxide on salicylic acid, jasmonate (and ethylene signaling cascades.** Schematic versions of salicylic acid (SA), jasmonate (JA), and ethylene (ET) signaling cascade. Biosynthetic enzymes are represented as gray ovals and signaling components are gray rectangles. Abbreviations in the jasmonate biosynthetic pathway are as follows: LOX, lipoxygenase; AOC, allene oxide cyclase; OPR, oxo-phytodienoate reductase; for the ethylene biosynthetic pathway: ACS, 1-aminocyclopropane-1-carboxylic acid synthase; ACO, 1-aminocyclopropane-1-carboxylic acid oxidase. Genes and their regulatory promoters are represented as open boxes. For details of signaling cascades, see main text. The known NO-regulated steps are indicated: green arrows indicating that NO has a promontory effects via induction of gene transcription; *S*-nitrosylative steps are indicated via a solid black bar and whether this promotes (green arrows) or inhibit a signaling (red bar) step. Note that for and ACO and ACS4, the effect of *S*-nitrosylation has yet to be determined.

Extensive characterization of plant interactions with pests and pathogens has allowed the major signaling networks governing biotic interactions to be elucidated (Davis, 1998; Preston, 2000; Quirino and Bent, 2003; Pieterse and Dicke, 2007). The hypersensitive response (HR) is effective mainly against (hemi)biotrophic pathogens and this form of defense is often associated with salicylic acid (SA; Mur et al., 2008a). SA acts via the induction of a plethora of defense genes, with the most-commonly described being acidic forms of pathogenesis-related protein (PR) genes such as *PR1* (Cao et al., 1994). The SA signaling pathway has now been extensively characterized (**Figure 1**). The translational activator NONEXPRESSOR OF PATHOGENESIS-RELATED PROTEINS1 (NPR1), localized in an oligomeric form in the cytoplasm (Fu et al., 2012) interact with the SA receptors NPR3 and NPR4 likely following redox changes at key cysteine residues that results in a monomeric NPR1 form which is translocated to the nucleus (Mou et al., 2003). Within the nucleus, NPR1 interacts with a range of TGA-class transcription factors which bind to TGACG motifs encoded within the promoters of SA-induced genes (Zhang et al., 1999).

Defenses against necrotrophic pathogens such as *Botrytis cinerea*, *Plectosphaerella cucumerina* (Berrocal-Lobo et al., 2002), and *Alternaria brassicicola* (Ton et al., 2002) have been linked to jasmonate and ethylene (hereafter referred to as JA and ET, respectively) signaling. Plant tolerance to insects is also strongly influenced by JA, so that there are similarities with resistance responses to necrotrophs (Howe and Jander, 2008). Both JA and ET signaling pathways have been exhaustively investigated and many good overviews are available (for example, Lin et al., 2009; Gfeller et al., 2010). Briefly, JA are lipoxygenase (LOX)-derived products of C18:3 acyl chains derived from phospholipids. JA is conjugated by JAR1 conjugatase to form (+)-7-iso-jasmonoyl-L -Ile (JA-Ile). JA-Ile interacts with the COI1 protein, a key part of a Skp-Cullin-F-box (SCF^COI^) complex which targets JASMONATE ZIM DOMAIN (JAZ) proteins (Chini et al., 2007). This interaction lead to the destruction of the JAZ repressors via the proteasome relieving their suppressive effects on a wide range of transcriptional activators MYC2, MYC3, and MYC4 (Chini et al., 2007; Fernandez-Calvo et al., 2011). The ET receptors ETR1, ERS1, ETR2, ERS2, and EIN4 are kinases which act as signaling repressors until ET binding occurs (Hua and Meyerowitz, 1998). The negative regulation occurs through the activity of putative MAP3K, CTR1 (Hua and Meyerowitz, 1998) which phosphorylates EIN2. EIN2 is a central component in ET signaling that in the phosphorylated form is located in endoplasmic reticulum. It is likely that dephosphorylation results in EIN2 translocation to the nucleus (Ju et al., 2012). Within the nucleus transcriptional activation involves components such as EIN2 and EIN3 regulating the expression of key transcription factors *ORA59* and *ERF1* (AP2/EREBP; Stepanova and Alonso, 2009). In the absence of ET, EIN3 is targeted by the SCF ligase, EIN3-binding F-box 1 and 2 (EBF1, EBF2) for destruction in the proteasome (Guo and Ecker, 2003; Potuschak et al., 2003; Gagne et al., 2004). As JAZ repressors also interact with EIN3, this would appear to be a crucial mechanism governing the JA/ET synergistic interactions (Zhu et al., 2011).

Under natural conditions plants are exposed to attacks from a range of pathogens and pests with a variety of infection strategies. Cross-talk between SA and JA/ET pathways allows the plant to divert resources to the most appropriate defense mechanisms (Pieterse et al., 2012). Thus antagonistic relationships are most often reported but synergistic SA and JA/ET pathway interactions also occur (Mur et al., 2005, 2006, 2008b). SA can suppress JA effects through the suppression of the JA biosynthetic enzymes LOX2 (Spoel et al., 2003) and allene oxide synthase (AOS, Laudert and Weiler, 1998). However, there are many points downstream of JA biosynthesis that are targeted by SA (Leon-Reyes et al., 2010). SA–JA cross-talk components include the protein kinase MPK4 (Petersen et al., 2000) and in particular the interplay of transcriptional regulators appears to play large roles in SA–JA antagonism.

Many antagonism mechanisms centre on the role of NPR1; as SA-mediated suppression of JA-mediated expression was abolished in *npr1-1* mutants (Spoel et al., 2003; Bargmann et al., 2009; Leon-Reyes et al., 2009; Pieterse et al., 2009). This antagonistic mechanism partially reflects an additional as yet poorly defined cytoplasmic role for NPR1 (Pieterse and Van Loon, 2004) but this does not seem to involve interference with SCF^COI1^-mediated targeting of JAZ proteins (Van der Does et al., 2013). However, the major SA–JA regulatory role for NPR1 appears to be nuclear-located. A transcription factor whose expression is partially NPR1-dependent is WRKY70 (Li et al., 2004). Over-expression of WRKY70 increased SA-dependent genes expression (*PR1*, *PR2*, and *PR5*) and suppressed JA-dependent defense gene transcription (*COR1* and *VSP1*); with anti-sense WRKY70 plant displaying opposite effects. Expression of WRKY70 is also partially induced by AtMyb44, which, interestingly, is induced via COI1 action suggesting a negative feedback step to modulate the amplitude of the JA response (Shim et al., 2013). This would represent a previously unsuspected role for an SA/JA antagonistic mechanism.

TGA-class transcription factors also appear to play a role in SA–JA interactions (Ren et al., 2008). Whilst many TGA factors regulate SA responsive gene expression, TGA2, TGA5, and TGA6 also induce JA and ET defense genes and crucially, also regulate SA-mediated antagonism (Zander et al., 2010). This suppressive mechanism includes a redox-regulated step catalyzed by glutaredoxins (GRX). GRX catalyze reactions whereby the oxidation of glutathione is coupled with the reduction of cysteine residues to influence protein stability and/or activity. GRX interacts with TGA2 and over-expression of GRX480 countered the induction of the *ORA59* promoter by EIN3 (Zander et al., 2012). In a parallel study, GCC box *cis* elements such as those found in the promoter of the JA marker gene *PDF1.2* were revealed as key sites through which SA–JA antagonism is effected. Focusing on two GCC-binding transcription factors it was found that ORA59 but not ERF1 was the key transcriptional target for the SA antagonistic mechanism (Van der Does et al., 2013).

Many other hormones interact with SA–JA/ET (Robert-Seilaniantz et al., 2011) but no comprehensive attempt has been made to integrate nitric oxide (NO) – a major defense signal – into the canonical SA–JA/ET interaction network. However, using plants displaying modulated expression of non-symbiotic hemoglobins (Hb) which oxidizes NO, we have demonstrated that NO plays an important role in both networks (Mur et al., 2012). Similar conclusions were advanced by Chun et al. (2012)who expressed mammalian NO synthase (NOS) in tobacco and observed increased resistance to pathogens via elevated SA and JA/ET defense gene expression.

## NITRIC OXIDE IN PLANT–PATHOGEN INTERACTIONS

Nitric oxide has emerged as a major player of plant resistance responses to biotrophic and hemibiotrophic pathogens influencing both basal defense and HR (Mur et al., 2005; Prats et al., 2005). Many studies on plant interactions with *Pseudomonas syringae* all indicate that NO is rapidly produced during the HR (Delledonne et al., 1998; Clarke et al., 2000; Mur et al., 2005) and perturbation of this NO generation has shown it clear contribution toward both cell death and other defense processes (Delledonne et al., 1998; Boccara et al., 2005; Mur et al., 2005; Prats et al., 2005).

Many groups with an interest in NO and plant defense are concentrating on thiol oxidation by NO, referred to as *S*-nitrosylation. *S*-nitrosylation comes about by the reaction of the oxidized form of NO, the nitrosonium ion NO^+^, which can electrophilically attack thiolate to produce *S*-nitrosylated thiols. This reaction can generate large pools of *S*-nitrosoglutathione (GSH + NO → GSNO + H^+^). GSNO itself can act as a nitrosylating agent and thus could act as a biochemical “memory” so that the effects of NO could persist after its generation has ceased or act as a mobile signal through which NO effects can be propagated throughout a plant as a component in systemic acquired resistance (Espunya et al., 2012). The reduction of GSNO pools is caused by the action of GSNO reductase (GSNOR) and predictably in AtGSNOR1 mutants GSNO levels have been observed to increase (Feechan et al., 2005). These AtGSNOR1 mutants were observed to exhibit compromised resistance to pathogens whilst over-expression of GSNOR increased defense against virulent pathogens. Such data suggested that it can be deleterious to form a GSNO “store” when NO is being generated.

If the thiol group belongs to cysteine residues of proteins, this results is an *S*-nitrosoprotein which can impact on protein function. A fascinating example of this is the control of reactive oxygen species (ROS) generation and cell death through protein *S*-nitrosylation. *S*-nitrosylation of cysteine 890 residue flanking the flavin-binding domain of the NADPH oxidase which is a major source of ROS generation during HR, suppressed both ROS generation and cell death (Yun et al., 2011). In addition, during the HR, *S*-nitrosylation and inactivation of two plastid-located peroxiredoxins (Prx; Sakamoto et al., 2003; Romero-Puertas et al., 2007) has been demonstrated. Prx can detoxify the highly reactive peroxynitrite ion which forms following the co-generation of $O_{2}^{-}$ and $NOxs(O_{2}^{-}+NO\to {ONOO}^{-})$and will generate hydroxyl radicals (ONOO^-^ + H^+^ → NO_2_ + OH). The toxicity of OH radicals is well-established and has been linked to PCD in animal systems so that through this Prx-mediated mechanism, NO acts with ROS to propagate cell death. This mechanism may appear to act in opposition to the apparently suppressive role of NO with NADPH oxidase but most likely reflected differential effects at different NO concentrations (Beligni and Lamattina, 1999; Garcia-Mata et al., 2003) and discrete roles at different stages in the development of the HR.

## NITRIC OXIDE AND SALICYLIC ACID SIGNALING

Following one of its first descriptions in plants (Delledonne et al., 1998), NO was immediately associated with SA-mediated events (Durner et al., 1998). Generation of NO through infiltration of mammalian NOS into plant tissues initiated SA-dependent gene expression (Durner et al., 1998). A comprehensive bioinformatic analysis of NO responsive promoters in *Arabidopsis* found that *cis* elements linked to SA responsiveness [ocs element-like sequences (OCSEs) and W-boxes] were prominent (Palmieri et al., 2008). We recently used transgenic over-expression and silencing of endogenous plant Hb in *Arabidopsis* to modulate NO generation in response to the hemibiotrophic pathogen *Pseudomonas syringae*, which demonstrated increased levels of SA accumulation in response to enhanced levels of NO and both were decreased in Hb over-expressing plants. Such observations placed SA “downstream” of NO generation but other data demonstrated that SA can modulate NO production. Thus, exogenous application of SA reduced NO production from tomato root tips (Gemes et al., 2011) and from stomata to initiate stomatal closure (Hao et al., 2010; Sun et al., 2010).

Studies of the mechanisms through which NO interacts with SA signaling appear to be particularly advanced (**Figure 1**). As already stated, the oligomeric status of NPR1 is essential to its action and *S*-nitrosylation of cysteine-156 has been shown to facilitate its oligomerization (Tada et al., 2008). Chemical reduction of this *S*-nitrosylated cysteine residue by SA-activated thioredoxin will promote monomer formation (Tada et al., 2008). Thus NO could be seen to play a paradoxical role: on the one hand initiating SA to promote NPR1 monomer formation and translocation from cytoplasm to nucleus, but on the other hand favoring oligomerization by initiating nitrosylation. These opposing roles are reinforced at other steps in SA signaling pathways. Thus, the positive effects of NO on SA pathway are further augmented by TGA1 *S*-nitrosylation that stabilizes the transcription factor and strengthens binding to cognate promoter sequences (Lindermayr et al., 2010). Against this are the effects of *S*-nitrosylation on SA-binding protein 3 (SABP3). SABP3 exhibits high affinity binding to SA and carbonic anhydrase activity. *S*-nitrosylation of SABP3 abolished SA binding and carbonic anhydrase activity (Wang et al., 2009). It may be assumed that this would have the same effect as silencing SABP3 gene expression which suppressed a HR elicited by *Pseudomonas syringae* pv. *tomato* (Slaymaker et al., 2002).

## NITRIC OXIDE AND ETHYLENE/JASMONIC ACID SIGNALING

Nitric oxide has often been reported to have a suppressive effect on ET signaling. Leshem and Pinchasov (2000)used laser photoacoustic detection to measure both NO and ET in ripening avocados and strawberry and noted that, on ripening, NO levels were reduced as ET increased. A mechanistic understanding of this interaction was provided by (Lindermayr et al., 2006, 2010). The Yang (methylmethionine) cycle produces *S*-adenosylmethionine (AdoMet) which is the methyl donor linked to the production of a range of metabolites including ET and also polyamines (Roje, 2006). Lindermayr et al. (2006)reported the *S*-nitrosylation of a key cysteine (Cys-114) within the active site of a methionine adenosyltransferase (MAT1; At1g02500) following the application of the NO donor – GSNO. *S*-nitrosylation by GSNO suppressed MAT1 enzymatic activity and also ET production. *S*-nitrosylation has also been noted in the ET biosynthetic enzymes 1-aminocyclopropane-l-carboxylic (ACC) synthase 4, although this has not been linked to a loss in enzymatic activity (Abat and Deswal, 2009).

Against such observations are our results which show the simultaneous generation of both NO and ET during a bacterially-elicited HR in tobacco (Mur et al., 2008a, 2009, 2012). We also noted that infiltration of a NO^+^ donor – sodium nitroprusside (SNP) – into tobacco leaves produced NO and also ET (Mur et al., 2005, 2008b). As SNP could induce ACC synthase expression (ACS), this seems to be one mechanism through which NO could boost ET production (Mur et al., 2008a, b). Similarly, the expression of mammalian NOS in transgenic tobacco increased ACC oxidase (the final enzyme in ET biosynthesis) and *ethylene-responsive element binding protein* (*EREBP*) expression (Chun et al., 2012).

Recently, we have also shown that NO positively contributes to elicit the production of jasmonates (Mur et al., 2012). Examining the transcriptional data provided by Palmieri et al. (2008)it can be seen that NO increases the expression of a range of JA biosynthetic genes. Thus, expression of LOX3 (At1g17420), 12-oxophytodienoate reductase 1, 2, and 3 (OPR1, 2, and 3; **Figure 1**), were induced by NO. Surprisingly, the expression of the intermediate JA biosynthetic enzyme – allene oxide cyclase (AOC) – is suppressed by NO (Palmieri et al., 2008) and AOC appears to be *S*-nitrosylated although an inhibitory effect has not been established (Romero-Puertas et al., 2008).

## INTEGRATING NO INTO SA AND JA/ETHYLENE PATHWAYS: THE CHALLENGES

To highlight the roles of NO in each pathway, it is useful to consider two differing scenarios (**Figure 1**). Upon infection with a (hemi)biotrophic pathogen NO will contribute to the initiation of SA biosynthesis through the relief of EIN3 repression of the *isochorismate synthase 1 *(*ICS1*) transcription (Chen et al., 2009). This relief possibly result from the suppression of ET biosynthesis through MAT1 nitrosylation so that EIN3 is degraded in the proteasome. SA will induce thioredoxins to reduce NPR1 protein to their monomeric form leading to their translocation to the nucleus. In the nucleus NPR1 will bind to TGA-class transcription factors. *S*-nitrosylation of TGA factors will increase affinity for their cognate promoters. Some TGA factors will bind to the ORA59 promoter to suppress both JA and ET-inducible genes. NPR1 will also contribute to the induction of WKRY70 to also suppress JA/ET expression. Another scenario is infection with a necrotrophic pathogen or attack by certain pests. JA biosynthesis occurs rapidly which is facilitated by NO-mediated induction of LOX3 and OPR1, 2, and 3. In addition, NO will induce ACS and ACO (1-aminocyclopropane-1-carboxylic acid oxidase) expression to increase ET biosynthesis.

This model is clearly too simplistic and poses a number of questions. When NO is generated, this should *S*-nitrosylate NPR1 to help maintain the oligomeric form which could suppress JA events (Pieterse and Van Loon, 2004; **Figure 1**). If so, how is JA biosynthesis achieved? Also, how can the induction of ACS by NO counter the effects of a MAT1 inhibition leading to a failure to provide methyl groups for ET production? Most importantly how is specificity conferred where SA and JA/ET pathways are simultaneous in play? One example of this is the *Pseudomonas syringae* pv. *phaseolicola* (*Psph*)-elicited HR in tobacco (Kenton et al., 1999; Mur et al., 2009).

 As we have recently pointed out (Mur et al., 2013) this could reflect subtle spatial–temporal separation in around sites of infection or insect grazing. Hb gene expression is regulated in a cell-specific manner and therefore could represent a way in which spatial regulation of local NO levels can be modulated by the plant in reaction to pathogens (Hebelstrup et al., 2013). Sub-cellular separation in signaling events should also be considered (Mur et al., 2013). Additionally, NO concentration is a key determinant of what regulatory step is employed. Thus, during the *Psph*-elicited HR in tobacco, NO production is rapidly induced but does not peak until 10–24 h following challenge whereas both SA and ET production is initiated at ~ 6 h following challenge (Mur et al., 2008b). During this phase it would be supposed that the positive effects of NO on SA and ET are paramount – as a low NO concentration effect – whilst JA production is suppressed via SA/JA antagonistic mechanisms possibly via *S*-nitrosylation of ACO. As NO production peaks it may be that the induction of JA-biosynthetic genes is initiated at high NO concentrations which could overcome any SA-antagonistic mechanism on this pathway. If the concentration-dependent mode for NO effects on SA/JA/ET signaling pathways is substantiated this suggest a major role for Hb. We have recently shown how reduced expression of Hb during the HR contributed to increased NO production (Mur et al., 2012) so this and NO generation mechanisms such as nitrate reductase (Gupta et al., 2011) could be important arbiters of the interplay between defense signaling cascades.

One way to address such complex interactions is suggested by the recent work of Windram et al. (2012). These authors sampled at 2 h intervals for 48 h to extensively describe transcriptomic changes occurring following attack of *Arabidopsis* by *B. cinerea* and then used Systems Biology modeling approaches to characterize key signaling hubs. Marrying this approach with careful measurements of signal generation patterns, would undoubtedly improve our understanding of the signaling interactions during plant defense and the place of NO in this network.

## Conflict of Interest Statement

The authors declare that the research was conducted in the absence of any commercial or financial relationships that could be construed as a potential conflict of interest.
